# Stars inside have reached outside: The effects of electronic dance music DJs’ social standing and musical identity on track success

**DOI:** 10.1371/journal.pone.0254618

**Published:** 2021-08-25

**Authors:** Hyeongseok Wi, Wonjae Lee

**Affiliations:** Graduate School of Culture Technology, Korea Advanced Institute of Science and Technology, Daejeon, Korea; University of Oxford, UNITED KINGDOM

## Abstract

The social standing of an artist provides a reliable proxy for the value of the artist’s product and reduces uncertainty about the quality of the product. While there are several different types of social standing, we focus on reputation among professional artists within the same genre, as they are best able to identify the artistic value of a product within that genre. To reveal the underlying means of attaining high social standing within the professional group, we examined two quantifiable properties that are closely associated with social standing, musical identity and the social position of the artist. We analyzed the playlist data of electronic dance music DJ/producers, DJs who also compose their own music. We crawled 98,332 tracks from 3,164 playlists by 815 DJs, who played at nine notable international music festivals. Information from the DJs’ tracks, including genre, beats per minute, and musical keys, was used to quantify musical identity, and playlists were transformed into network data to measure social positions among the DJs. We found that DJs with a distinct genre identity as well as network positions combining brokerage and cohesion tend to place higher in success and social standing.

## Introduction

“Cultural markets,” such as the music market, have traditionally confronted uncertainty issues in terms of product quality [1] because these markets elude mechanistic and systematic evaluations and call for a complex mixture of formalized knowledge, informal reference, experience, taste, and personal judgment [2]. However, Podolny [3] suggests that social standing may be a useful measure to evaluate artistic products in markets with high uncertainty. A market of singularities cannot function without evaluation by experts [4]; therefore, while there are several different types of social standing, the artist’s reputation within a group of peers who create competing products merits attention. This is because, given the limited access to expertise, reputation among professional artists provides consumers with a more reliable reference.

This study seeks to establish that an artist’s social standing is closely associated with both their musical identity and their social position. First, we ascertain the artist’s musical identity. In this dynamic cultural market, musical identity cannot be put into distinct, well-defined categories. Instead, it comprises complex and nuanced combinations of characteristics that assign artistic value to certain targets, resulting in some having a higher standing than others. Therefore, we assume that musical identity is a quantifiable measure that reflects the ability to distinguish and capture a niche within a specialist market.

Second, because social standing is conferred through social affiliations [5], we focus on the social position of the artist by analyzing the relationships between artists within a social network [6]. The social relations of artists are personal and based on homophily [7]. The artistic relationships that artists choose to build lead to ideas and opportunities and offer the artists a better chance to create conventions through which to address uncertainty [7–9].

To achieve these goals, this study targets the electronic dance music (EDM) DJ market, a growing specialist market in the music industry. DJ/producers play a more dominant role in the production system than is the case with the general music market, where the musical production system is finely segmented. They desire to be highly acclaimed musically and socially to gain a competitive advantage within a group of similar artists. Moreover, DJs are evaluated in terms of their playlist sets, which include tracks by other DJs along with their own. Therefore, the social position of a DJ is established through the DJ’s selection of tracks. As relational-oriented artists, DJs directly reveal social relations with other DJs by citing them, effectively showing deference by including the tracks of other DJs within their own playlists.

Social standing in the EDM market has been discussed by Janosov et al. [10], who analyzed the DJ Mag Top 100 chart to identify the emergence of mentorship dyads between recognized and less experienced DJs. These musical collaborations are a social force maintaining the centering of each musical herd around the leaders. In line with Janosov et al.’s work, our study explores how DJs attain social standing. We focus on two properties: musical identity and social position. In particular, we attempt to identify how artists with high social standing strategically develop a niche and take the lead in an emerging cultural market.

## Literature review

### Social standing of professionals in the cultural market

Given their variety and complex indices of quality, cultural products are difficult to assess in terms of value [11]. Individual works are inimitable and difficult to compare, and no clear criteria exist to estimate genuine or precise quality levels. The uncertainty associated with these products has remained prominent in cultural studies within the fields of economics and sociology, targeting cultural markets including art [12], fashion [13], film [14], and music [15]. These studies generally focus on identifying useful and reliable parameters to assist with the evaluation of cultural products and anticipate which products will be successful.

Economists suggest that the value of a cultural product can be analyzed in the same way as other products that are costly to produce and whose values are determined by the interplay between supply and demand [7]. However, this approach tends to oversimplify the nature of the cultural market, which is inherently much more complex.

Sociologists, on the other hand, have interpreted the valuation of cultural products as involving collective action by the participants in the market. Becker’s novel work “Art World” [16, 17] and Bourdieu’s “Artistic Field” [18] stipulate that cultural products should be examined in terms of their social interaction. This assumes that social mechanisms lead a product to acquire high artistic and financial value [19]. Salganik and Watts [20] applied this approach specifically to cultural markets and found that significant social influence led to the success of a product in an artificial music market. Yogev [7] asserted that exchange relations and active participation in a market help to socially define high-quality art products. Pachucki and Breiger [9] also insist that the cultural market is a dynamic process of meaning creation influenced by the structure of associated social networks, directly accounting for the sociological perspective and emphasizing the importance of social context.

From this point of view, professional creators (e.g., artists, musicians, and writers) play pivotal roles in attaching value to cultural products, because they have the best understanding of the creation of cultural products, including those that have high artistic complexity or require intensive expertise. These reputable professionals, therefore, receive attention and bestow meaning through their actions. Thus, their leverage in the market is relatively high and provides a reliable reference for consumers. The cultural market cannot function properly without the evaluation of these experts [4].

The process of evaluation and acknowledgement of a cultural product is based on socially constructed judgments of quality [6]. Therefore, recognition by professional colleagues is a crucial criterion for success within a cultural market. It is a strong competitive advantage that cannot be easily acquired and does not easily change [21]. Beckert [11] emphasizes that the reputation of experts affects price formation in the art market. Further, Zhao and Zhou [22] found that multiple social-standing indicators considerably influence wine sales outcomes. Rossman et al. [14] analyzed the film industry and found that the number of participants in a film with a high social standing positively correlated with the number of Oscar nominations the film received. Additionally, Lynn et al. [23] showed that the choices of those who are considered mainstream in the music market affect the extent to which consumers find the music to be likable.

To become a recognized artist within a certain market, one might attempt to bolster one’s social standing as a member of a professional group. Social standing is acquired through social connections and interdependencies between influential artists of similar or different styles who are active in the field. Therefore, artists seek opportunities for competition and collaboration to help build their reputations. Eventually, they attract analogous creators and adherents, and form groups that share similar tastes. Based on homophily, these connections operate as pillars, supporting the uniqueness of the group. Such groups can establish new styles and conventions, and even create trends.

To determine the means by which social standing may be acquired in the music market, we analyzed the playlist sets of EDM DJs. In conjunction with the dramatic growth of the electronic music market, EDM DJs have transcended the conventional role of a disc jockey, which was to simply mix consecutive tracks [24], to become major music composers and performers. Their playlist sets receive attention and are considered to be aural artworks that provide a blueprint or musical traveling guide for international music festivals. These DJs painstakingly and devotedly create playlists by selecting tracks from among their own compositions and those of other DJs. They endeavor to satisfy their audiences’ preferences by displaying their sophisticated taste via track citations and their ability to compose via their mixing skills. Citations of other DJs’ tracks in their playlists are often aimed at gaining social acknowledgement. Thus, EDM DJs are relation-oriented artists who depend on professional colleagues and groups to gain recognition as musical artists. This serves as a powerful parameter of musical and commercial evaluation for general consumers in the market, who eagerly seek out influential artists to follow.

In this study, we regard the number of track citations in playlist sets as a type of success among EDM DJs. We propose two factors that affect how this success functions as a sign of social standing within this professional group: musical identity and social position.

### Artistic identity of professionals

Traditionally, identity is seen as an individual’s characteristic determined by specific attributes. It is considered a solid, intrinsic single factor, or a discrete category comprising a classification system. A market develops standards or means that can be used to categorize individuals. Accordingly, identity is formed through the adherence of individuals to these criteria in their interactions with others [25].

Categorical labels such as rigid genre classifications are useful for measuring identity. This process of assigning identity facilitates interactions among professionals [26] and acts as a cultural convention by which artworks can enter a market and play a significant role in structuring taste and consumption behavior [27, 28]. Therefore, it prescribes the range of cultural product offerings [29] and enables critics to compare and evaluate works [30].

However, recent studies argue that in markets, identities are more complex than commodities because they involve networks of interrelated constructs of multiple attributes [31]. The building of an identity involves a consistent process of reconstruction and reinterpretation of oneself in the environment [32]. In this sense, identity is mobile—a process and not a thing, a becoming and not a being [33]. It can be interpreted strategically as an experience of self-in-process.

Sociologists have sought to use quantifiable measures to examine identity in its complexity and individuality. For example, Askin and Mauskapf [15] quantified musical characteristics such as the tempos and harmonies of popular Billboard tracks to find evidence of musical patterns formed as shared identities. Lena and Peterson [8] analyzed the musical identities of jazz musicians into more detailed hierarchical sub-genres. Therefore, the multivocal and complex identities can be measured quantitatively.

A multivocal identity may be associated with multiple roles or groups. Successful avoidance of a categorical label means that one’s identity may be accepted in positive roles outside of that labeled category. This preserves flexibility not afforded by those with more narrowly defined identities that induce commitments to restricted lines of action [34]; simple identities constrain future opportunities. Jensen and Kim [35], for example, describe the benefit of multivocal identities in the opera market: balancing divergent styles of opera can lead to a broad appeal to different audiences and less competitive pressure within a restricted single category. Further, Jaffe [36], analyzing the commercial film industry, reported that the construction of a new identity involves not selecting and fitting into a single existing genre, but deliberately combining aspects of diverse genres.

However, a complex identity can also incur social penalties in the form of dis-attention or outright rejection for not conforming to prevailing social cognitive frames. Attempting to broaden one’s identity to include multiple and diverse roles incurs the risk of being devalued for defying the conventional boundaries that guide valuation. For example, Hsu [37] asserts that films spanning multiple genres are less appealing to both critics and general audiences.

Accordingly, the preferred type of identity differs depending on the niche width or resource space of the artist’s market [35]. Niche width theory suggests that a complex, generalist identity is advantageous in a volatile environment but disadvantageous if environmental resources are stably concentrated in a single category where specialist identities prevail [38]. On the other hand, resource partitioning theory asserts that both generalists and specialists can coexist in some environments. They are not necessarily in competition because the niches overlap only to a limited extent [39].

Zuckerman et al. [34] emphasize that generalist and specialist identity claims do not conflict with one another, but instead apply to different contexts. The question of whether simplicity or complexity is more advantageous relies on one’s location in reference to a point at which a multivalent identity is construed as no identity at all [34]. van Venrooij and Schmutz [40] assert that genre ambiguity has a negative effect within a commercial subfield, but not in an artistic subfield. Additionally, Noh and Tolbert [41] segmented art museum visitors in detail from professional critics to general consumers, and found that all categories of visitors responded differently to museums with complex versus simple identities.

Although the EDM DJ culture is a recent phenomenon, the EDM market can be seen as a specialist rather than a general music market. Specifically, the EDM market exhibits an uncommon reliance on DJs, who are the dominant element in the musical process. The credibility of professional DJs rests on their specialized taste and knowledge of music, and, therefore, on knowledge boundaries. The EDM market has a dynamic classification system in which categories are often mixed, and new categories frequently emerge.

In such a specialist market, the role of an expert is crucial in forming the preferences of general consumers. This is an example of leading through a distinct identity related to expertise. For example, van Venrooij and Schmutz [40] found that music albums with complex identities, as measured by genre ambiguity, generally receive negative reviews from critics. However, Goldberg et al. [42] argue that clear categorical boundaries convey specialized knowledge and increase the perceived cultural depth and artistic value of a product. Similarly, Noh and Tolbert [41] found that professional critics’ reviews showed a clear preference for single-category types of art museums. Considering this consistent preference for a focused identity in professional groups within a cultural market, we formulate the following hypothesis:

*H1*. *Tracks composed by DJs with focused identities will receive more citations*.

### Social position of professionals

Social position is a crucial factor in gaining recognition from professional colleagues in a cultural market [43]. Artists do not stand alone. Talent and status naturally lead to network connections through mentorships, sponsorships, collaborations, and citations, leading artists to interact with their colleagues [44]. This results in further inspiration and the creation of more cultural products. This process is an indirect indicator of the recognition of artistic value in markets in which uncertainty related to evaluation is relatively high. Clearly, it is conceptually difficult to disentangle this process from a social context. Therefore, the strategic ability to build social interactions can influence an artist’s value by playing a role in the process of conforming to or differentiating oneself within or across niches [37, 45]. This consequently facilitates social cohesion and differentiation through exchanges of tastes and styles, which helps one not only to establish methods by which to participate but also to form and define boundaries. Collective interactions within these boundaries form the conventions of distinctive genres and subcultures [46]. To capture the opportunities and constraints inside these boundaries, artists must be aware of the embedded advantages of their social positions.

The importance of social position in a cultural market was first explored in Howard Becker’s “Art World” [16]. Becker’s theoretical framework of the types of artists depicts creative work as collective action centered on interactions and collaborations, and reveals how relationships shape success and enhance status. Since the publication of this framework, sociologists have agreed that notable artistic works characterized by creativity, innovation, and popularity are outcomes of collaborative interactions that are influenced by social positions.

For example, Slavich and Castellucci [47] show that creative professional chefs strategically position themselves via mentoring relationships to gain recognition from critics for their novel work. Crossley [48] similarly highlights the importance of networks between key social collaborators in the punk music scene in Manchester. Further, Uzzi and Spiro [49] analyzed artistic networks on Broadway, and concluded that relationships lead to the exchange of resources and support the dissemination of influential information to external audiences.

There are two types of social positions with benefits in a market: the brokerage position and the cohesive position. Specifically, Podolny [21] likens the properties of the two structural positions to pipes and prisms, respectively. Pipes are channels through which information and other resources can pass from one actor to another in the network. Prisms mirror the product or producer so that it can be distinguished by consumers. Both types of social positions have structural advantages that can be strategically parlayed into a reduced level of uncertainty in a dynamic market.

The brokerage position features weak external ties that control the flow of information and provide opportunities and ideas [50, 51]. Occupiers of this position tend to receive knowledge from multiple sources and interpret information from diverse perspectives. By recombining and transferring knowledge, individuals whose positions extend across social divides and connect otherwise unconnected nodes of the network are better positioned to detect non-redundant, valuable knowledge [52].

Analyses of the brokerage position in cultural networks focus on brokering or boundary-spanning roles, emphasizing the unique informational benefits of these positions. For instance, Pinheiro and Dowd [53] reported that jazz musicians’ critical and financial success is correlated with conversance in multiple genres, which can lead to beneficial associations with other recognized musicians. By analyzing musician networks, Giaquinto et al. [54] revealed that individuals occupying brokerage positions are open-minded and display crossover styles. Furthermore, Lingo and O’Mahony [55] found that cultural producers in brokerage positions draw on a range of relationships to integrate project contributors’ ideas while supporting creative outcomes.

Unlike the brokerage position, the cohesive position involves strong ties, which can amplify trust and facilitate power based on these strong attachments [56]. By developing reciprocity norms and self-enforcing governance mechanisms, a cohesive position can foster high levels of cooperation and greater volumes and depths of information sharing, thus affording an incentive to conform to norms [57]. Additionally, this position can help one efficiently gain the visibility necessary for valuable recognition in a given context.

In the cultural market, the advantage of the cohesive position is often closely related to the locality and homophily of one’s taste or style. For example, using ethnographic methods, Uzzi [58] documented how cohesive networks supported richer information flows among New York garment designers and manufacturers. Grandadam [59] highlighted the importance of participating in and following the conventions of a cohesive network for success as a jazz musician. This is because convention eases the transfer of information and knowledge, particularly tacit and complex knowledge, which is often difficult to transmit. By analyzing rap music collaborations among artists of similar rap styles, Smith [60] found that dense network clusters based on a local community are a crucial factor in gaining recognition.

The choice of brokerage or cohesion appears to involve a trade-off, as the conceptual properties of the two positions are at odds structurally. The advantages of either type of position or the optimal marginality between the two positions has been investigated by considering the conditions of the market. For instance, Uzzi and Spiro [49] investigated cohesion among Broadway musical creators and its impact on the success of their musicals. They found that cohesion had a positive impact up to a certain point, but began to impede creativity when it became too prevalent. Cattani and Ferriani [61] assert that an unbiased intermediate position is key to creative performance in the film industry because it allows for a balance between the benefits of brokerage and cohesion. Furthermore, Phillips [62] analyzed jazz musicians playing actively in international cities to show that both a highly disconnected cohesive city (Sydney) and a highly connected brokerage city (New York) independently lead to successful, stylistically creative performances of jazz music.

The advantages of the two social positions are also complementary [63], given that it is difficult to realize the value of having many loose ties unless combined with well-established cohesive ties that provide ready access to collaborative alignment, leading to novel information and ensuring trust. Fleming et al. [64] assert that creativity and success are correlated with the social cohesion of collaborators who work within multiple organizations and have connections with external contacts. In a cultural market, Crossley [48] emphasizes the importance of enthusiastic focus groups and mediators who can serve as bridges between groups, based on the finding that both were essential elements in the prosperity of the 1960s punk music scene in England.

By examining a hybrid position that contains both benefits from brokerage and cohesive positions for a synergistic effect, researchers have suggested compatibility between the two network properties [44, 65]. A combination of internal cohesive ties and external brokerage ties generates the advantage of a “small world” network [49]. This argument is not new; however, it was previously framed from the perspective of a macro-network [49, 66] rather than an individual collaborating with other individuals, who in turn have both internal and external ties [67]. Burt [63] describes this concept as a structural autonomous actor who belongs to a densely interconnected group of colleagues, but also has bridging ties beyond them. Similarly, Tortoriello [43] argues that the brokerage position is most beneficial when the bridging tie is a Simmelian tie [68], indicating strong cohesive ties among connected groups. Additionally, Vedres and Stark [69] introduced the concept of a “structural fold,” which refers to a network position of inter-cohesion that provides both familiarity and diversity for innovative actions.

The hybrid position has taken the spotlight in specialist markets, where the complexity and variability of evaluations are relatively high because, to minimize uncertainty in the market, competitive participants adopt both pipe and prism strategies. Ter Wal et al. [70] point out that in specialist markets, where a cohesive advantage is indispensable, the expanded openness afforded by the brokerage position is crucial to outperform others. Further, Baum et al. [65] analyzed investment syndicate ties in the context of Canadian banks, and found better firm performance to be linked with the hybrid position, combining ready access to diverse information sources through brokerage and integration of information through cohesion. Sullivan and Ford [71] also found that venture capital firms embedded in networks with denser clustering levels but shorter path lengths are more inclined to engage in exploratory learning.

The dynamic nature of the EDM DJ market makes it a specialist market that is separately segmented to minimize overlapping resource spaces [39]. DJs deliberately attempt to structure hybrid network positions to enhance their social standing. New styles and fresh artists break onto the EDM scene without warning, creating new value criteria. Therefore, DJs enter networks at the periphery, eventually becoming more sought after as partners, developing brokerage ties with other parts of the network, and leveraging their success toward hybrid positions as they develop their abilities and visibility over time.

To gain a better understanding of the role of recognized DJs, the term “anchor tenant” has been used as a substitute for the concept of the hybrid position [72]. An *anchor tenant* refers to a large department store in a retail shopping mall that creates demand externalities for other shops. Large department stores with recognized names generate mall traffic, which indirectly increases the sales of lesser-known stores. Similarly, as hubs in their music market network, market-leading DJs improve the absorptive capacity and enhance the localization of artistic knowledge spillover. Hybrid positions have the competitive advantage of affecting group behavior and allowing one to lead through strategic choices, dictating the direction of the market. Therefore, DJs with high social standing gain stability in a rapidly changing market through strong cohesive ties while simultaneously leading local colleagues through open artistic innovations and creativity acquired via brokerage connections with other subgroups. This leads to the following hypothesis:

*H2*. *Tracks composed by DJs in hybrid network positions will receive more citations*.

## Data and measurements

DJ network and musical identity data were collected for 62 time windows from 2013 to 2016. DJs’ citation network was constructed using R based on the DJs’ playlist data, and the musical identity data for each DJ were calculated based on track information, including genre, beats per minute (BPM), and musical key. Once this information was in place, the dynamics of the DJ networks and musical identities were analyzed through negative binomial, fixed-effects models using Stata 14.

### Data sources

#### 1001Tracklist.com

1001Tracklist.com is an open-source playlist database that lists DJs’ playlist information. The database includes the following data: (1) the date and event of the playlist; (2) the composition of the playlist, including the tracks’ titles and artists, and the order played; and (3) views and likes of the playlist.

After reviewing over 500 annual EDM festivals from 2013 to 2016, using a web crawling module created in Python, we chose tracks from nine notable annual EDM festivals held during this period for our analysis. A total of 3,164 playlists by 815 DJs from 62 events were chosen. The selection criteria for the nine festivals were as follows. (1) Only world-famous festivals with over 50,000 attendees and approximately 200 DJs performing were chosen. Each festival cast almost the same DJs who perform around the world. (2) Only festivals lasting 2 to 6 days were chosen to assign only one event to a single time window. The approximate number of visitors and performing DJs at each festival for each analyzed year is presented in Table 1.

**Table 1:** Approximate numbers of visitor and performing DJs at the selected festivals.

Festival	2013	2014	2015	2016
Ultra Music Festival	330,000/400	165,000/230	165,000/240	165,000/220
Electric Daisy Carnival	345,000/240	400,000/240	400,000/240	400,000/290
Tomorrowland	180,000/240	360,000/280	180,000/350	180,000/430
Creamfields	150,000/160	150,000/170	150,000/180	200,000/180
Nature One	64,000/90	72,000/95	65,000/90	65,000/95
Mysteryland	60,000/150	60,000/100	100,000/130	100,000/105
Electric Zoo	107,000/140	100,000/120	150,000/100	83,000/125
Tomorrowworld	140,000/220	150,000/190	180,000180	-
Stereosonic	50,000/90	60,000/100	50,000/100	60,000/100

The nine festivals from which data were collected for this study are as follows (location, total approximate number of visitors): (1) Creamfields (worldwide, 150,000); (2) Electric Daisy Carnival (worldwide, 1,545,000); (3) Electric Zoo (USA, 440,000); (4) Mysteryland (worldwide, 320,000); (5) Nature One (Germany, 266,000); (6) Stereosonic (Australia, 220,000); (7) TomorrowLand (Belgium, 900,000); (8) Tomorrowworld (USA, 650,000); and Ultra Music Festival (worldwide, 825,000).

To identify the social standing of scene-leading DJs, we applied the concept of a citation network analysis to the DJs’ playlist data. A citation network analysis represents a collection of scholarly papers as a network to reveal the importance of a particular paper using citation counts and other structural features. Likewise, with the DJs’ playlist data, we were able to construct a music citation network. The frequency of tracks cited by DJs signifies the importance of the track in the professional EDM music scene.

#### Beatport.com

Beatport is an influential online music store that targets EDM DJs. Many popular international DJs are registered with this service. The site functions as a market for DJs who want to legitimately acquire tracks to construct playlists or remixes. On Beatport, each DJ has an individualized webpage containing a full list of their output, and the DJs can also sell tracks or albums using the service.

Beatport provides basic information about tracks, including the release date, musical genre assigned by the composer from among 28 genres provided in Beatport, BPM or tempo, and the key or harmonic scale. This information is essential for other DJs who want to purchase and download the track for their own use, especially when constructing playlists, given that the style and skill set of the DJs allows them to creatively mix tracks based on the aforementioned information. The data can be acquired through the corresponding official data/API, which is open to the public. Consequently, we collected 98,332 pieces of track data from 815 DJs who provided their playlists. From this track data, we used the genre, BPM, and key of each track to calculate the musical identity of each DJ. Beatport offers 28 categorized genres that cover every existing style of EDM. When a DJ releases a track, they must select the genre of the track from among the 28 listed genres. Beatport also offers the BPM and key of the tracks, which is the most essential feature information for DJs to arrange tracks in a playlist. BPM ranges from 60 to 235, and the key corresponds to each of the 12 major and 12 minor musical scales comprising notes A–G with sharps and flats. Therefore, a total of 132 BPM values and 24 keys were used in the analysis.

### Construction of the network data

**Fig 1:**
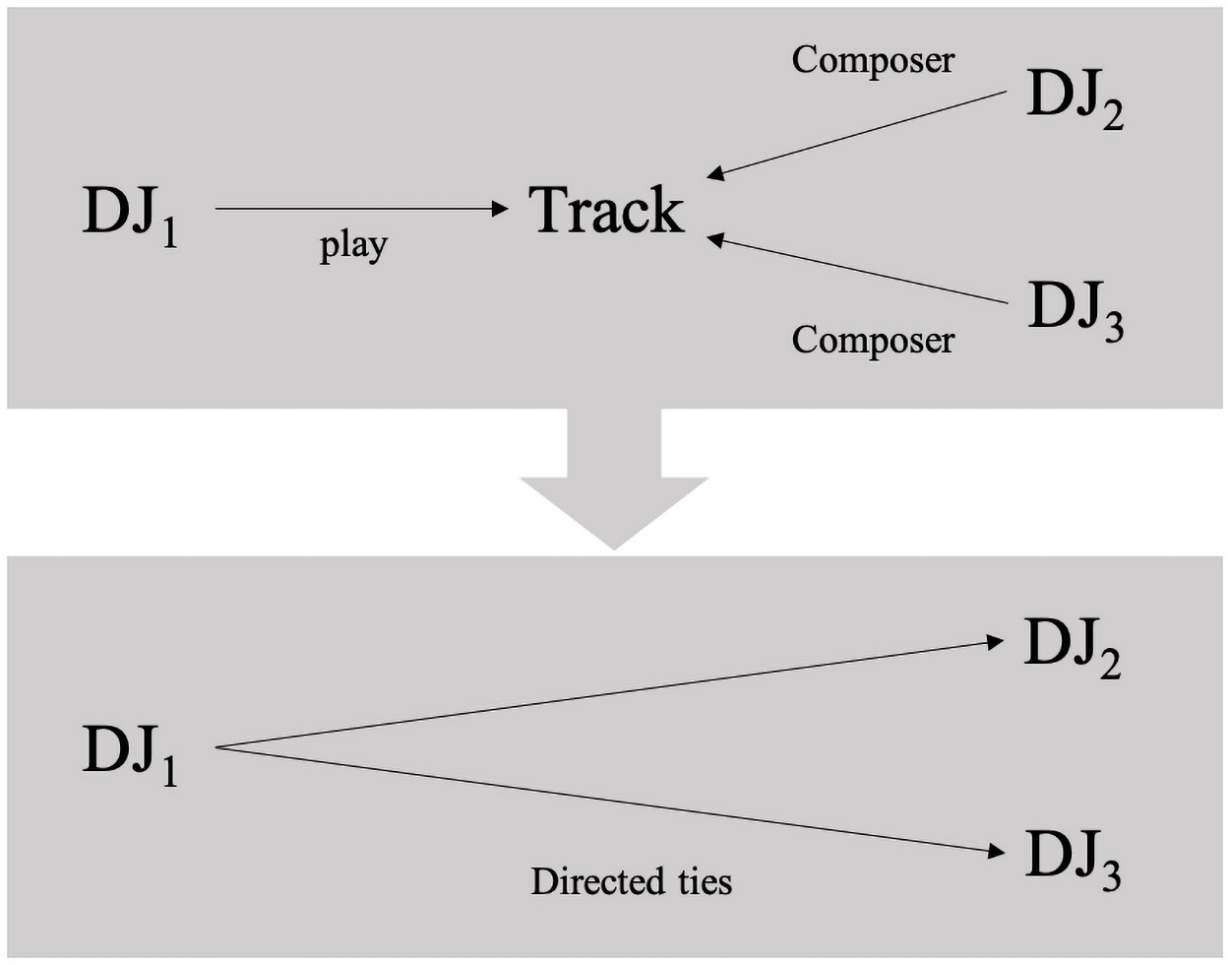
Construction of the DJ citation network.

Social networks are represented as graphs composed of nodes (actors) and edges (ties). In this study, a node represents a DJ. The directed edge between two nodes indicates that the DJ cited another DJ. To consider the different levels of appreciation given to the cited DJs, we used weighted edges. A “weighted edge” accounts for the “cost” of the edge, or the value of the edge from one node to another. For example, if DJ_1_ plays the tracks of DJ_2_ multiple times in a playlist, it indicates a greater appreciation of DJ_1_ for DJ_2_ compared to other DJs that are cited only once by DJ_1_. As shown in Fig 1, DJ networks were constructed based on the playlist set data. When a playlist by DJ_1_ includes tracks composed by DJ_2_ and DJ_3_, we consider DJ_1_ as having interacted with DJ_2_ and DJ_3_. We constructed 62 time windows of DJ interaction (play) networks based on the occurrence of a festival event. The timespans between the events are regular, at roughly one month, which is suitable for a longitudinal analysis. As a result, a total network of 817 DJs and 81,688 ties was constructed. There was an average of 220±75 DJs per festival and 1339±907.85 edges per time window. The popularity of each track was calculated based on how often it was played during each time window.

### Applying track data as musical identity data

Music and artists are classified according to their musical styles. Musical style is defined as a mixture of melody, rhythm, mood, harmony, and instrumentation [73]. Generally, it is categorized into genres that are based on definable musical features and provide a set of shared expectations [37]. However, sociologists argue that genres should not be defined merely by their musical characteristics. Genre divisions also include social considerations, such as the historical, cultural, and economic background of the artists and listeners, which structure musical production, consumption, and social boundaries [74].

EDM is an umbrella term that covers a heterogeneous group of musical styles. As in many types of music, electronic music has also evolved into various sub-genres that take into account different periods, regional, and social dimensions [74]. DJs creatively build their musical identities to differentiate or associate within and outside of genres through the composition of tracks and playlists. Therefore, genre classification is an informative factor to be considered in ascribing a musical identity to an artist.

The BPM and key of the track are also fundamental parameters in EDM [75]. BPM is a rhythmic indicator of a track, and helps the DJ to construct rhythmic patterns, as tempo is an important feature that can help distinguish the genre of a track [76], often considered above melody and harmony [77]. BPM is crucial because DJs determine the order of a playlist to control the rhythm throughout the sequence of the tracks. “Mixing,” or marking smooth transitions between tracks, is an important skill associated with musical continuity.

Along with BPM, key is a tonal indicator that is a primary consideration in mixing. Tonality in EDM has been discussed in terms of composing techniques for DJs and is considered a core component along with BPM [78]. To mix harmonically, DJs must know the musical keys (scales) of the records they play. Matching the key allows the tracks to combine naturally. The ability to mix harmonically provides DJs full control over the energy of the room because they can select keys that boost or lower the energy of the crowd.

In Beatport, each track is accompanied by the genre, BPM, and key data. These three types of data were collected from tracks to determine the musical identity of the DJs. For example, the track data included for “Shocker” by Tiesto are Trap (genre), 140 (BPM), and F# min (key). For the longitudinal analysis, the three types of data from each track were individually summed one year prior to each time window. Therefore, we crawled data from 2012 to 2016. For instance, DJ Tiesto played at Tomorrowland 2013, which was held in September 2013 and was the twelfth event (T12) in the longitudinal dataset. We aggregated DJ Tiesto’s track data from September 2012 to September 2013 in Beatport. Therefore, the most recent musical identity of DJ Tiesto is reflected according to his work output. For example, during the tenth event (T10), DJ Tiesto composed 97 tracks in a total of 6 genres (Electro House:47, Trance:44, House:3, Electronica/Downtempo:2, Dubstep:1, Future Bass:1). We aggregated the squared ratio of each genre count to measure genre consistency. The number of composed tracks is different for each DJ in each time window. Therefore, we calculated the ratio of each genre count to the total number of composed tracks. However, the sum of the ratio of genre counts equals one. Thus, we used the sum of the squared ratios to compare the values of each DJ’s track data. The genre consistency of DJ Tiesto during the eleventh event (T11) was (47/97)^2^+(44/97)^2^+(3/97)^2^+(2/97)^2^+(1/97)^2^ +(1/97)^2^ = 0.432. In this way, we calculated the BPM and key consistencies in the eleventh event as 0.237 and 0.08, respectively.

### Dependent variable

#### Number of track-citation counts

Citation counts for each track were calculated for each time window. A high citation count indicates that the track was cited numerous times by other DJs. Possible explanations are: (1) the track is a signature track representing a certain musical style, (2) the track is a well-known hit, and/or (3) the track is a desirable source for manipulation. For these reasons, these tracks are meaningful for professional DJs within the scene.

### Independent variables

#### Musical identity

Essentially, the musical identity of a DJ is expressed through the tracks they compose. To determine the DJs’ musical identity based on data from the music, each DJ’s track features were analyzed to measure whether the extent of their musical identity was clear and distinctive. We calculated the ratio of each track data to the total number of composed tracks for each time window. We then used the sum of the squared ratios to represent the consistency of musical identities ranging from 0 to 1. For example, if the genre consistency of DJ *i* is high, the genre identity of DJ *i* lies firmly within a specific genre. This indicates that the DJ has a focused musical identity. However, if genre consistency is low, DJ *i*’s musical identity involves the exploration of a variety of genres.

#### Social position

Betweenness centrality [79] is a general network measure of brokerage that measures the extent to which each individual actor falls between other actors in a network. This measure is associated with the role of brokers or gatekeepers, who control resources and information flows. As pipes, actors with higher betweenness centrality occupy a structurally advantageous position with regard to finding new knowledge or utilizing innovation diffusion because they connect different groups [63].

Theoretically, the betweenness centrality of node k is measured based on the number of times that node i reaches node j via the shortest path passing from node k [80]. Hence, the presence of a node on the shortest path between any two other nodes in a network increases the node’s betweenness centrality, and in turn, the node has greater control over the two non-adjacent nodes [81]. Hence, the betweenness centrality of node k (betweenness centrality_k_) is defined as follows:

$$Betweenness\;centrality_{k}=\underset{i\;\neq k\;\neq j}{\sum }\frac{\sigma_{ij}(k)}{\sigma_{ij}}$$ (2–1)

where σ_*ij*_ is the total number of shortest paths from node *i* to *j*, and σ_*ij*_ (k) is the number of shortest paths from node *i* to node *j* that contain node k.

The clustering coefficient, also called cliquishness, refers to the number of triangles with nodes at each corner in a given undirected graph. This is used to measure the level of clustering in the network. In other words, it is the likelihood that two neighbors of a node are also connected to each other [82]. As a prism, DJs with high clustering coefficients tend to cluster with other DJs, resulting in a tightly knit sharing of artistic taste and a high number of connections among the DJs. These DJs may compose playlists of similar styles by means of internal referencing among the cluster members. Watts and Strogatz [83] define the clustering coefficient based on a local clustering coefficient (LCC) for each node within a network. The local clustering coefficient is defined as follows:

$$lcc_{i}=\;\frac{number\;of\;triangles\;connected\;to\;node\;i}{number\;of\;triples\;centered\;on\;node\;i}$$ (2–2)

where the numerator is the number of instances in which three nodes, including node i, are all connected to each other. The denominator represents the number of sets of two edges that are connected to node *i* (triples).

### Control variables

We controlled for DJ-level dyadic constraint variables that may be confounded by the independent variables. These include the following: (1) the total number of citations and released tracks of the DJs, (2) the concentration of degrees (described below), and (3) the degree of reciprocity of the control variables.

We included the total number of times that DJ *i* citied others (out-degrees) and the total number of times that DJ *i* was cited by others (in-degrees). We also included the number of tracks released by each DJ in each time window.

The concentration of degrees refers to the variance of each DJ’s ties. Both in-degrees and out-degrees were calculated. High in-degree concentration signifies citation of a DJ *i* by other DJs who are loyal and dedicated to *i*. Low in-degree concentration signifies citation of a DJ *i* by various DJs. Out-degree concentration is the opposite of in-degree concentration. High out-degree concentration signifies many citations of particular DJs. Low out-degree concentration signifies the citation of tracks of various DJs. Considering [*a*_*ij*,*t*_] as an adjacency matrix of a time window *t*, the in-degree and out-degree concentrations are as follows:

$$in-degree\;concentration_{j}=\;\overset{n}{\underset{i=1}{\sum }}{\left(a_{ij.t}/\overset{n}{\underset{i=1}{\sum }}a_{ij.t}\right)}^{2}$$ (2–3)

$$out-degree\;concentration_{i}=\;\overset{n}{\underset{j=1}{\sum }}{\left(a_{ij.t}/\overset{n}{\underset{j=1}{\sum }}a_{ij.t}\right)}^{2}$$ (2–4)

The content and degree of resource flow invested through social ties are asymmetric in dyadic relationships. Reciprocity is a measure of the likelihood that vertices in a directed network will be mutually linked in two time series. High in-degree reciprocity signifies that a DJ steadily reciprocates citations by other DJs at previous events. High out-degree reciprocity signifies that a DJ is steadily cited back by those previously cited by him. Both in-degree and out-degree reciprocity values were calculated for each DJ. If DJ *i* cited DJ *j*’s track in the previous time window (*t*-1) and DJ *j* cites DJ *i*’s track in the present time window (*t*), the notation of in-degree and out-degree reciprocity is denoted as follows:

$$in-degree\;reciprocity_{j.t}=\;\overset{n}{\underset{i=1}{\sum }}(a_{ij.t}/a_{ji.t-1})$$ (2–5)

$$out-degree\;reciprocity_{i.t}=\;\overset{n}{\underset{j=1}{\sum }}(a_{ij.t}/a_{ji.t-1})$$ (2–6)

## Methodology

Because the data are time-series and cross-sectional, we employed the negative binomial, fixed-effects regression model derived by Hausman et al. [84] to estimate the effects of these variables on the number of track citations. The model utilizes a negative binomial distribution, which is a generalization of the Poisson regression model. However, it overcomes the limitations of the Poisson distribution, which assumes the variance to be equal to the mean. It has been acknowledged as a more appropriate distribution in working with count data, such as traffic-related fatality counts.

Let *y*_*it*_ be the number of citations in a time window t for track *i*. We assume that *y*_*it*_ bears a negative binomial distribution with an expected value *λ*_*it*_ and a variance given by *λ*_*it*_(1+*θλ*_*it*_). The parameter *θ* reflects over-dispersion. This model has the advantages of accounting for heterogeneity between tracks over a given time window and factoring out over-dispersion parameters. This means that a lack of information regarding other factors that may influence the dependent variable does not result in biased estimates [85]. The negative binomial fixed-effects regression model is expressed as follows:

$$\begin{array}{c} \text{log}\lambda_{it}=\alpha_{i}+\beta x_{it}\\ i=1,2,\ldots N\\ t=1,2,\ldots T \end{array}$$ (2–7)

where *λ*_*it*_ is the expected number of citations of track *i* in a given time window *t*. The independent variable *x*_*it*_ is followed by a one-time lagging window behind the dependent variable. β denotes the maximum likelihood estimation of *x*_*it*_. Parameter *α*_*i*_ is the effect of tracks, and the effect of events is included in *x*_*it*_. As can be seen, we adjusted for both track and event fixed effects. By doing this, we ensured that neither stable traits of tracks nor those of events account for the effect of the key covariates. This is important because preferential sorting based on a DJ’s musical identity and social positions is plausible for tracks with stable and unobserved qualities. For example, this adjustment controls for cited tracks of different lengths owing to the construction of playlists. Similarly, the adjustment controls for festival stages that bear divergent environments. Track and event fixed-effects remove hidden variable biases that are based on unobserved characteristics.

Given that the unit of analysis is a track, the model includes the standard deviation for each variable except for the dependent variable, because tracks are not composed exclusively by individual DJs. In fact, 72% (5,102 tracks) of the 7,087 tracks were composed by as many as 11 DJs. The unit of the independent and control variables was the DJ. Therefore, we calculated the mean and standard deviation of each variable.

For the longitudinal analysis, the dependent variable is measured in the time window at *t*+1. Therefore, all the independent and control variables are lagged by one-time window at *t*. For a better understanding of our model, The descriptive statistics for the variables are shown in S1 Table, which shows that the correlation coefficient between the dependent and independent variables is fairly low (Average Betweenness Centrality: 0.007, Average Clustering Coefficient: 0.011, Citation per Track_t_: 0.238).

## Results

The results for the three estimated fixed-effect negative binomial models are presented in Table 2. Models 1 and 2 include either social position or musical identity as variables to test the significant association of each independent variable with social standing. Model 3 includes all variables, showing a significant association of both social position and musical identity with social standing. As the standard errors of the key variables exhibit little difference between the reduced and full models, we consider Model 3 to offer a more secure basis for interpretation.

**Table 2:** Negative binomial regressions predicting the number of citations per track _t+1_.

Variable		Model 1	Model 2	Model 3
Social Position	Average Betweenness Centrality _t_	27.513***		22.879***
		(7.027)		(7.081)
	Standard deviation of Betweenness Centrality _t_	-11.800		-2.439
		(11.112)		(11.034)
	Average Clustering Coefficient _t_	0.166***		0.136***
		(0.036)		(0.036)
	Standard deviation of Clustering Coefficient _t_	-0.542***		-0.359***
		(0.082)		(0.082)
Musical Identity	Average Genre Consistency _t_		0.137*	0.131*
			(0.058)	(0.058)
	Standard deviation of Genre Consistency _t_		-0.503***	-0.443***
			(0.132)	(0.132)
	Average BPM Consistency _t_		0.376***	0.358***
			(0.055)	(0.055)
	Standard deviation of BPM Consistency _t_		-1.319***	-1.281***
			(0.125)	(0.125)
	Average Key Consistency _t_		-0.394***	-0.385**
			(0.085)	(0.085)
	Standard deviation of Key Consistency _t_		-0.456	-0.445**
			(0.158)	(0.158)
Control Variable	Average Number of Indegrees _t_	-2.802	-1.025	-3.314
		(1.995)	(1.888)	(2.005)
	Standard deviation of Number of Indegrees _t_	-32.453***	-24.475***	-23.865***
		(4.312)	(4.239)	(4.313)
	Average Number of Outdegrees _t_	-1.950	1.805	-1.644
		(1.577)	(1.017)	(1.583)
	Standard deviation of Number of Outdegrees _t_	-6.255*	-7.825***	-6.703*
		(2.440)	(1.754)	(2.431)
	Average Indegree Concentration _t_	-0.096**	-0.169***	-0.130***
		(0.034)	(0.031)	(0.034)
	Standard deviation of Indegrees Concentration _t_	-0.603***	-0.471***	-0.393***
		(0.072)	(0.071)	(0.073)
	Average Outdegrees Concentration _t_	0.016	-0.041	-0.032
		(0.106)	(0.106)	(0.106)
	Standard deviation of Outdegrees Concentration _t_	-0.680***	-0.578**	-0.576**
		(0.199)	(0.197)	(0.197)
	Average Indegree Reciprocity _t_	-0.088	-0.101	-0.106
		(0.096)	(0.096)	(0.096)
	Standard deviation of Indegree Reciprocity _t_	-0.054	-0.010	-0.025
		(0.149)	(0.150)	(0.150)
	Average Outdegree Reciprocity _t_	0.396	0.352	0.369
		(0.206)	(0.205)	(0.206)
	Standard deviation of Outdegree Reciprocity _t_	-1.151***	-1.236***	-1.205***
		(0.347)	(0.347)	(0.347)
	Average Number of released tracks _t_	0.001***	0.000	0.000
		(0.000)	(0.000)	(0.000)
	Standard deviation of Number of released tracks _t_	-0.011***	-0.008***	-0.008***
		(0.001)	(0.001)	(0.001)
	Citations per track _t_	0.176***	0.180***	0.181***
		(0.003)	(0.003)	(0.003)
Constant		-2.150***	-2.108***	-2.147***
		(0.061)	(0.065)	(0.066)
Track Fixed Effects		Yes	Yes	Yes
Event Fixed Effects		Yes	Yes	Yes
N		288,491	288,491	288,491
Log Likelihood		-75194.13	-75069.92	-75051.79
*χ* ^2^		8839.10(p < .000)	9049.63 (p < .000)	9096.30 (p < .000)
(p-value)		(0.000)	(0.000)	(0.000)
The Number of IDs		7,087	7,087	7,087
VIF		1.76		

Robust standard errors are in parentheses;

*** p<0.001,

** p<0.01, and

* p<0.05.

The fit of the models is presented by Wald *χ*^2^, which is statistically significant in all three models (Model 1: *χ*^2^ = 8839.10, p = .000; Model 2: *χ*^2^ = 9049.63.10, p = .000; Model 3: *χ*^2^ = 9096.30, p = .000). We measured the variance inflation factor (VIF) to be 1.76. The VIF of the independent variables is less than 2, which indicates no problematic multicollinearity between the independent variables.

Consistent with our expectations, the results show that both the average betweenness centrality and average clustering coefficient of the DJs are positively associated with the reputation of the tracks they composed. This suggests that DJs composing more popular tracks tend to be constrained in their local networks but are also connected to other local clusters. The significant and negative coefficient of the standard deviation of the average clustering coefficient suggests that the tracks are more appreciated when the composing DJs are more homogeneous in terms of social positions.

The average in-degree concentration and its standard deviation exhibits a negative association with the number of citations per track across all three models. This is evidence of a wide variety of connectivity among DJs who composed highly reputable tracks. It also serves as an indication that DJs with a similar degree of connectivity are more likely to produce more popular tracks. On the other hand, the average out-degree concentration is insignificant, which suggests that the popularity of the tracks is associated with the popularity of the composing DJ, not with their musical references.

**Fig 2:**
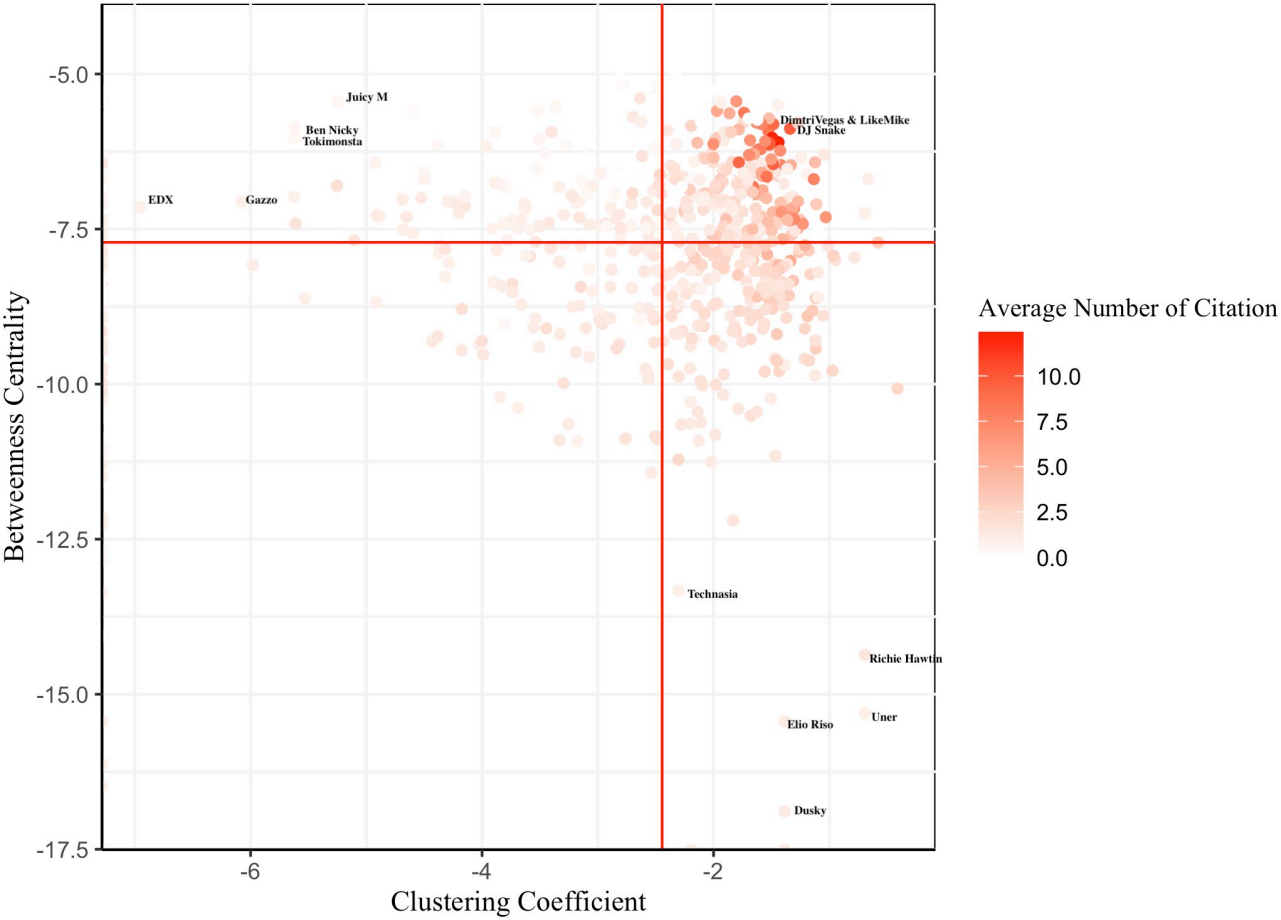
Distribution of DJs by social positions. The axes are the social position variables. We take the log on both social position variables: Betweenness Centrality and Clustering Coefficient. The red lines are the medians of both variables.

**Fig 3:**
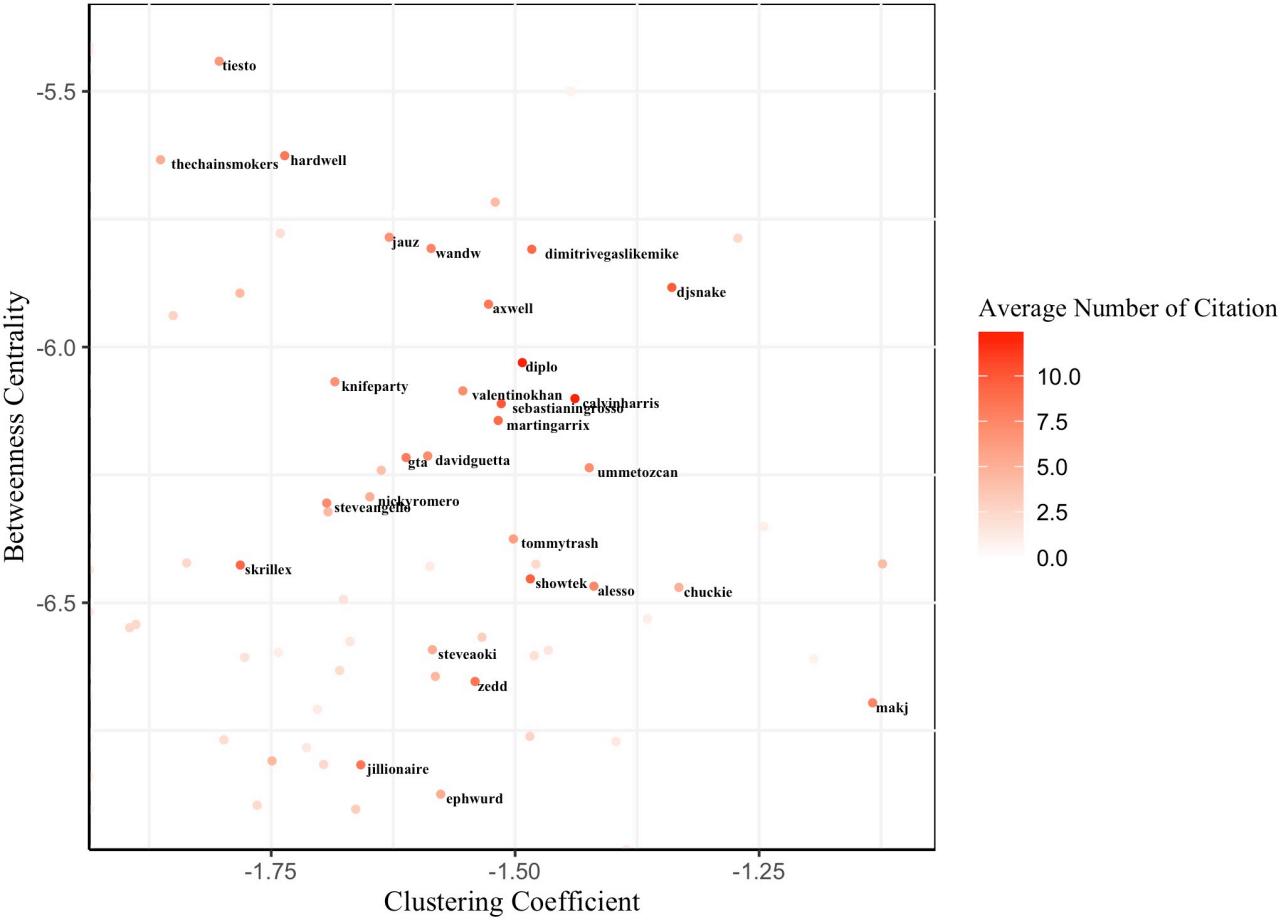
Hybrid social positions of 30 most cited DJs. This figure is a magnified cut-out of the upper right quadrant of Fig 2. The name-tagged DJs are well-known representatives of each musical style.

Fig 2 displays the distribution of all DJs by taking the log of both social position variables, which shows the hybrid position of DJs with higher social standing. Fig 3, the magnified cut-out of the upper right quadrant of Fig 2, shows the 30 DJs who are most frequently cited by other DJs. These are well-known DJs in the EDM industry and are representatives of each affiliated musical category. We verified that most of them are constantly selected for the DJ Mag Top 100, the most prestigious annual event for EDM DJs [10]. For example, *Dimitri Vegas&LikeMike*, and *DJ snake* are among the most highly ranked DJs in the DJ Mag Top 100 during the time of analysis. The name-tagged DJs in the upper left quadrant of Fig 2 are the notable DJs in typical brokerage positions. In particular, *EDX* is well known for remixing popular tracks from various genres. He was nominated for a 2019 Grammy Award for Best Remixed Recording. The name-tagged DJs in the lower right quadrant of Fig 2 are notable DJs in typical cohesive positions. All of them are Techno house DJs who are relatively closed to other genres and are exclusive to each other. In particular, *Richie Hawtin* is one of the symbols in Techno who is well versed with Techno music.

The focused musical identity variables are measured by the degree of consistency of each DJ genre, BPM, and key distribution of the tracks. Consistent with our hypotheses, the results show that the average genre and BPM consistency exhibit a significant positive association with the number of citations per track. Considering the importance of the rhythmic basis in dance music, the genre and BPM consistency represent the DJ’s focused identity, which may constitute legitimating exemplars or resource competitors with regard to others [40]. Therefore, the focused identity can have a strong influence on institutionalizing a specific genre, which enables one to dominate locally as an internal musical representative. The significant and negative coefficients of the standard deviations of both variables indicate that tracks composed by DJs with similarly focused identities are more appreciated.

However, key consistency is negatively associated with an increased number of citations. High harmonic scale variance suggests that DJs with high social standing are able to compose appealing tracks of diverse moods. To enhance the dramatic transition between tracks, DJs painstakingly compose tracks that fit the keys to control the energy of the track or playlist. These DJs tend to secure a genre and BPM range to highlight their focused musical identity, even though the harmonic scale varies.

**Fig 4:**
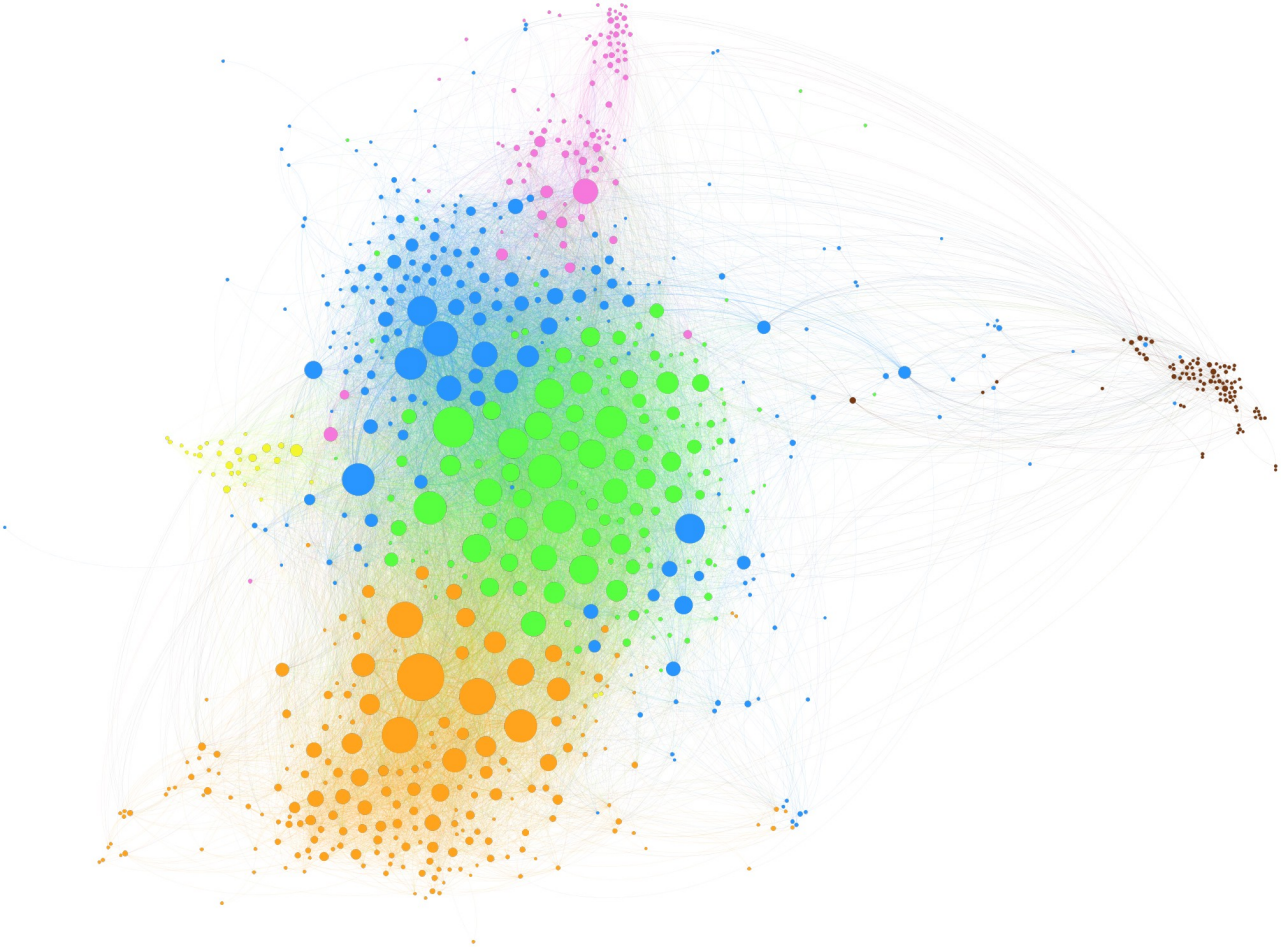
Total citation network of DJs. The figure shows the total citation network of the DJs by adding up the temporal networks from each time window. The node size represents the total number of citations by other DJs. The edge weight is presented by the thickness of the edge.

**Fig 5:**
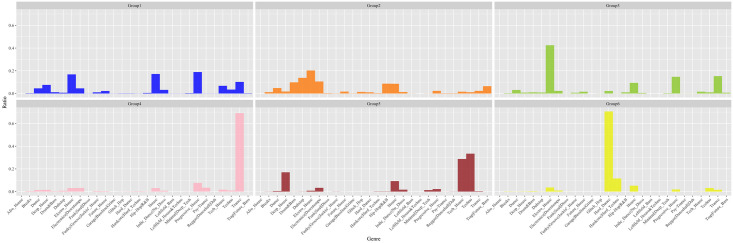
Genre distribution of detected groups. A total of 6 groups are detected using the fast greedy optimization algorithm: Group 1 (blue/Progressive House), Group 2 (orange/Dubstep, Drum, & Bass), Group 3 (green/Electro House), Group 4 (pink/Trance), Group 5 (brown/Techno, Tech House) and Group 6 (yellow/Hardcore, Hard Dance).

**Fig 6:**
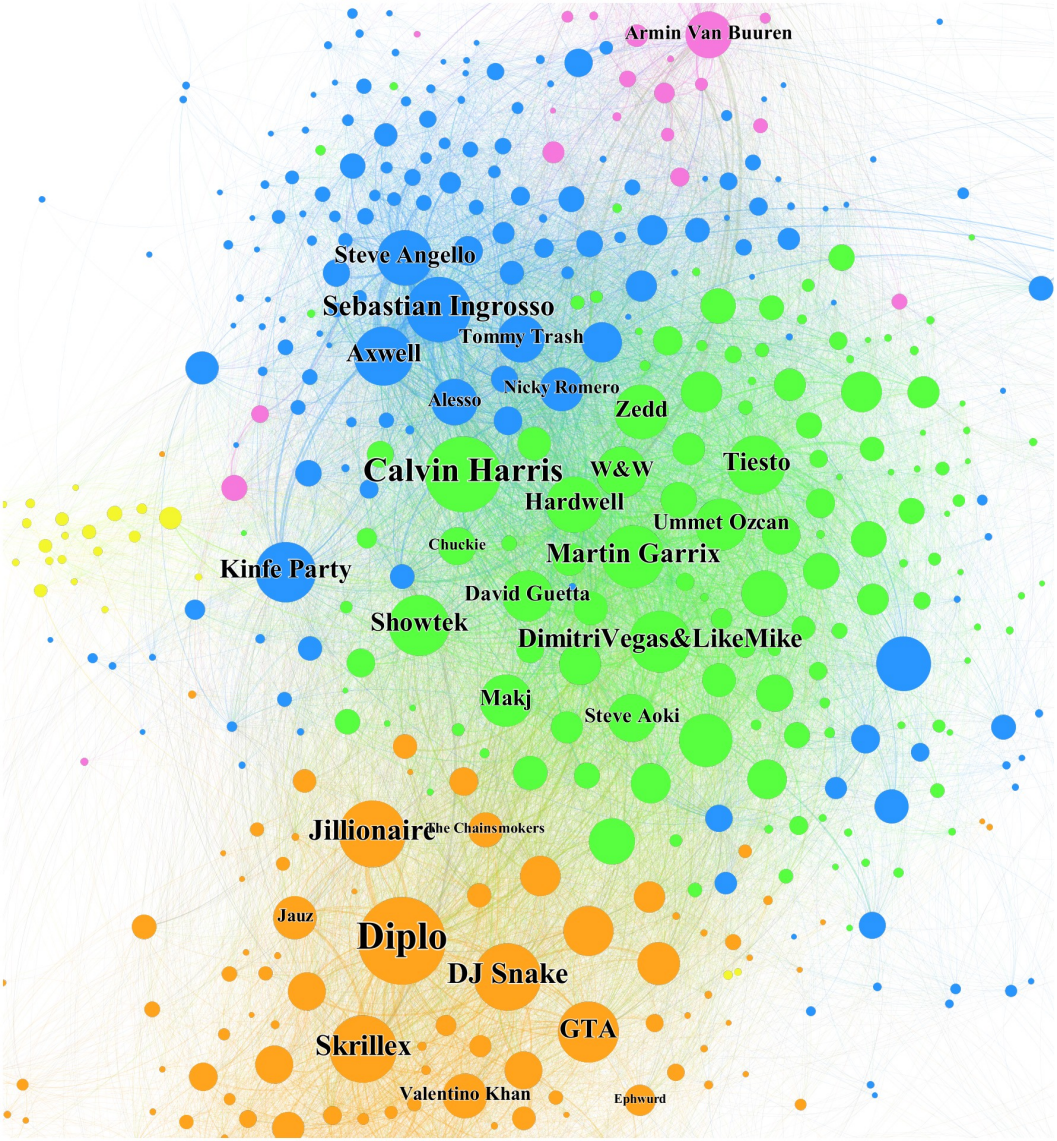
Location of 30 most cited DJs in the citation network. The figure is an enlargement of the central part of Fig 4. The name-tagged DJs are the same as in Fig 3.

Using Gephi, Fig 4 shows the total citation network of the DJs by summing the temporal networks from each time window. We used the fast greedy modularity optimization algorithm [86] to place the DJs into local groups. Fig 5 shows the distribution of genres within each group, making it clear that the DJs are grouped in terms of similarity of musical style. The centrally concentrated DJs in Fig 4 signify the strong interconnectedness between DJs pulling each other to central positions within each group. In contrast, techno Group 5 was isolated at the outer position. We also tagged the names of the 30 most cited DJs in a hybrid position, as shown in Fig 6. Most of the DJs were from the central positions within each group. In particular, the musical styles of Groups 1, 2, and 3 are known to be comprehensive and readily accessible to DJs in other groups. We confirmed that the 30 most cited DJs account for 29.6% of all external edges in other groups. They averaged 41.3% external edges, while other DJs averaged 28.2%.

Each recognized DJ adheres to a distinctive identity while playing the role of a broker with external ties, since in many contexts brokers are more likely to exhibit multivocal identities and a corresponding crossover to different genres [44]. Although cultural omnivorism is key to conveying high status [87], the results here suggest that while acting as brokers, DJs within each subgroup tend to maintain their particularized identities. The remix culture prominent in EDM provides a convincing explanation for this phenomenon. Because constructing playlist sets represents a DJ’s main specialty, the ability to creatively combine a variety of tracks using one’s own artistic style is crucial. To showcase their remix skills, DJs skillfully select tracks to maximize the displays of their talent. Recognized DJs prefer to select tracks from other genres, borrowing from existing contexts and creating new reinterpretations while drawing upon their own musical backgrounds. The greater the appreciation these DJs show for an affiliated musical category, the greater the possibility of receiving favorable feedback from members of that category.

### Robustness checks

The results are derived from longitudinal network data and are insufficient to circumvent the problem of endogeneity, which occurs when the DJs comprising the networks and the number of track citations are inseparable. Network effects are needed to guard against the possibility of confounding factors that affect both the position in the interaction networks and the number of track citations [88]. To address this issue, two separate robustness checks were conducted: a randomization test and a coarsened exact matching (CEM) [89].

One technique for testing the independent effect of networks on social standing is to compare the difference between the original network coefficients with the effects of a randomly wired network whose size and density are identical to those of the original network [90]. If the difference is significant, we can conclude that the effect of the observed networks is not trivial.

The network randomization test begins with the generation of the number of ensembles of an observed network. Controlling for size and density, we generated ensembles by rewiring an actual network. The total number of nodes and edges were fixed, preserving the weights of the edges and degree distribution [86]. Once the ensembles were generated, a predefined function (betweenness centrality and clustering coefficient) was computed for each. The coefficients of this function draw a distribution for a null hypothesis. The original coefficient from the actual data was then compared against the distribution from ensembles to test the statistical significance of the original results.

We created 200 randomized ensembles of the original interaction networks. From the 200 ensembles, we obtained 200 unique βs. βs are the maximum likelihood estimations of independent variables from the models resulting from the ensembles. These βs are assumed to be drawn from a Gaussian distribution whose mean and standard deviation are equal to those of the ensembles. We then obtained a z-score for the original β in the distribution of 200 βs. Generally, if the z-score of the original β is greater than 1.96, we can say with 95% confidence that the relationship is significant.

In the randomization test for our model, the z-scores for betweenness centrality and clustering coefficient were 2.19 and 2.92, respectively. This indicates that the original βs are much higher than those of the randomized ensembles, and that the correlation between network centrality and the dependent variable cannot be entirely attributed to unobserved confounding factors.

As a second check of robustness, we included the same covariates entered previously, and performed coarsened exact matching (CEM) [91] using our data. In doing so, we sought to distinguish the effects of the covariates from the confounding effects.

This produced a substantial reduction of 48% in the imbalance. Without event fixed effects and the average number of released tracks and its standard deviation, the CEM models offer support for our main hypothesis. The results are shown in Table 3.

**Table 3:** Negative binomial regressions predicting the number of citations per track _t+1_ (CEM sample).

Variable		Model 1	Model 2	Model 3
Social Position	Average Betweenness Centrality _t_	20.289**		18.553**
		(6.735)		(6.771)
	Standard deviation of Betweenness Centrality _t_	-4.101		-4.831
		(11.643)		(11.694)
	Average Clustering Coefficient _t_	0.087*		0.086*
		(0.035)		(0.035)
	Standard deviation of Clustering Coefficient _t_	0.311***		0.277**
		(0.088)		(0.089)
Musical Identity	Average Genre Consistency _t_		0.198***	0.198***
			(0.058)	(0.058)
	Standard deviation of Genre Consistency _t_		0.765***	0.731***
			(0.150)	(0.150)
	Average BPM Consistency _t_		0.154**	0.146**
			(0.054)	(0.054)
	Standard deviation of BPM Consistency _t_		0.041	0.004
			(0.139)	(0.140)
	Average Key Consistency _t_		-0.253**	-0.237**
			(0.080)	(0.080)
	Standard deviation of Key Consistency _t_		-0.725***	-0.710***
			(0.173)	(0.173)
Control Variable	Average Number of Indegrees _t_	-23.578***	-22.035***	-22.958***
		(1.828)	(1.731)	(1.830)
	Standard deviation of Number of Indegrees _t_	16.232***	12.233**	12.593**
		(4.699)	(4.680)	(4.755)
	Average Number of Outdegrees _t_	-0.951	1.944	-0.616
		(1.511)	(1.001)	(1.515)
	Standard deviation of Number of Outdegrees _t_	7.869**	8.101***	7.859**
		(2.685)	(1.969)	(2.692)
	Average Indegree Concentration _t_	-0.411***	-0.445***	-0.406***
		(0.033)	(0.031)	(0.033)
	Standard deviation of Indegrees Concentration _t_	0.940***	1.017***	0.941***
		(0.078)	(0.076)	(0.080)
	Average Outdegrees Concentration _t_	0.004	-0.018	0.003
		(0.104)	(0.104)	(0.104)
	Standard deviation of Outdegrees Concentration _t_	0.200	0.182	0.156
		(0.210)	(0.209)	(0.210)
	Average Indegree Reciprocity _t_	-0.091	-0.085	-0.093
		(0.097)	(0.097)	(0.097)
	Standard deviation of Indegree Reciprocity _t_	-0.030	-0.046	-0.010
		(0.178)	(0.179)	(0.179)
	Average Outdegree Reciprocity _t_	0.360	0.331	0.356
		(0.201)	(0.200)	(0.201)
	Standard deviation of Outdegree Reciprocity _t_	-0.585	-0.563	-0.556
		(0.358)	(0.357)	(0.357)
	Citations per a Track _t_	0.148***	0.147***	0.147***
		(0.003)	(0.004)	(0.004)
Constant		-1.418***	-1.463***	-1.508***
		(0.030)	(0.036)	(0.039)
Track Fixed Effects		Yes	Yes	Yes
Event Fixed Effects		No	No	No
N		183,326	183,326	183,326
The Number of IDs		6,480	6,480	6,480

## Conclusion

This study investigates how social standing is attained within a professional group of artists whose members play a key role in evaluating their artistic products in the EDM market. These artists provide reliable indicators to consumers, reducing the amount of uncertainty associated with products. Acquiring social acknowledgment within a professional group is an effective way to ensure the quality of products they produce and, thus, of a strong reputation.

The findings of this study suggest that musical identity and social position are two crucial factors that affect social acknowledgment by a professional group. First, the results demonstrate that focused musical identity is correlated with social standing among EDM DJs. The EDM market is an emerging niche market that is constantly developing and differentiating new styles and genres. It includes artists who establish the value criteria and demarcate the categorical space into separate identity positions reflecting artistic forms of a similar type [92].

Second, this study focuses on the two advantages of two types of social positioning, brokerage and cohesive, which can effectively reduce uncertainty in the market. The results show that DJs taking a hybrid position, combining elements of both brokerage and cohesion, have higher social standing. This hybrid position is the most advantageous position for controlling new opportunities and inflows of resources and for utilizing them. Unlike existing studies that divide the merits of the two positions into a dichotomy, this study follows the practice of recent studies that show that the two positions can generate synergy in a complementary manner.

This study attempts to reveal the relationships among EDM DJs who are accustomed to using the tracks of other DJs intuitively. Moreover, we also examined the musical identity of artists according to the musical features of the tracks, which is essential information for DJs as they delicately select tracks.

Our result for a specialist market in which the strategic role of a scene leading artist is critical is consistent with prior research. Despite the strategic importance of professional artist groups within the cultural market, the interior social mechanisms have not yet been thoroughly investigated, which may be because they can be affected by market conditions or players. It is also difficult to analyze the significant attributes of a cultural product that is related to success in the market in terms of social relations as the social relations affecting the products are intangible and ambiguous.

However, as an aural product, we analyzed the EDM DJs’ playlist data which intuitively reveals the nature of the internal dynamic relations. There is a possibility that it might be associated with other quantifiable properties. The results here emphasize the strategic role of the focused identities of hub DJs and of collaborative social connections between different subgroups operating through symbiotic relationships.

We revealed that artists with high social standings interdependently share resource spaces even when they prosper in different niches in specialist markets, such as the EDM market. However, we also found that the unique case of Techno group 5 provides evidence that the structural properties can vary depending on musical genres. Therefore, to further expand research in this area in the future, we will focus more on the nature of social relations within each musical group to reveal the how the strategies for success can be differentiated according to the musical genres. Moreover, we plan to associate the playlist data with other variables such as artistic longevity. Lastly, we will test whether our hypotheses and results apply to the musical identities and social positions of professional artists in other cultural market domains.

## Supporting information

S1 Table Descriptive statistics of variables. (N = 288,491).
(DOCX)

S1 File The data for the negative binomial fixed-effects regression model. (CSV)
