# Supplementary material for: Stars inside have reached outside: The effects of electronic dance music DJs’ social standing and musical identity on track success
Source: PLoS One. 2021 Aug 25;16(8):e0254618. doi: 10.1371/journal.pone.0254618 (PMC8386830; doi:10.1371/journal.pone.0254618)
Supplement: S1 Table — (N = 288,491). (DOCX) [file pone.0254618.s001.docx]

## **S1 Table. Descriptive statistics of Variables. (N = 288,491)**

| **Variable** | **Mean** | **SD** | **Min** | **Max** | **1** | **2** | **3** | **4** | **5** | **6** | **7** | **8** | **9** |
| --- | --- | --- | --- | --- | --- | --- | --- | --- | --- | --- | --- | --- | --- |
| 1. Citation per a Track _t+1_ | .149 | .747 | 0 | 34 |  |  |  |  |  |  |  |  |  |
| 2. Citation per a Track _t_ | .143 | .740 | 0 | 34 | .238 |  |  |  |  |  |  |  |  |
| 3. Average Number of Indegrees _t_ | .007 | .007 | 0 | .056 | -.002 | .141 |  |  |  |  |  |  |  |
| 4. Standard deviation of Number of Indegrees _t_ | .001 | .003 | 0 | .039 | .024 | .099 | .334 |  |  |  |  |  |  |
| 5. Average Number of Outdegrees _t_ | .006 | .010 | 0 | .105 | -.009 | .041 | .266 | .122 |  |  |  |  |  |
| 6. Standard deviation of Number of Outdegrees _t_ | .003 | .007 | 0 | .075 | .028 | .072 | .154 | .440 | .372 |  |  |  |  |
| 7. Average Indegree Concentration _t_ | .391 | .314 | 0 | 1 | -.003 | -.099 | -.642 | -.187 | -.264 | -.141 |  |  |  |
| 8. Standard deviation of Indegrees Concentration _t_ | .073 | .153 | 0 | .707 | .021 | -.008 | -.115 | .469 | -.020 | .276 | .150 |  |  |
| 9. Average Outdegrees Concentration _t_ | .027 | .079 | 0 | 1 | -.008 | .000 | .040 | .022 | .174 | .049 | -.106 | -.009 |  |
| 10. Standard deviation of Outdegrees Concentration _t_ | .012 | .048 | 0 | .707 | .017 | .027 | .039 | .231 | .058 | .274 | -.053 | .208 | .387 |
| 11. Average Indegree Reciprocity _t_ | .034 | .106 | 0 | 1 | -.004 | .032 | .190 | .085 | .581 | .218 | -.166 | -.011 | .115 |
| 12. Standard deviation of Indegree Reciprocity _t_ | .019 | .071 | 0 | .707 | .019 | .057 | .132 | .323 | .268 | .585 | -.109 | .173 | .054 |
| 13. Average Outdegree Reciprocity _t_ | .013 | .043 | 0 | 1 | -.002 | .035 | .277 | .093 | .181 | .082 | -.190 | -.024 | .056 |
| 14. Standard deviation of Outdegree Reciprocity _t_ | .007 | .028 | 0 | .707 | .020 | .069 | .195 | .359 | .121 | .293 | -.122 | .141 | .029 |
| 15. Average Betweenness Centrality _t_ | .001 | .002 | 0 | .029 | -.007 | .049 | .444 | .158 | .807 | .294 | -.361 | -.042 | .159 |
| 16. Standard deviation of Betweenness Centrality _t_ | .001 | .001 | 0 | .021 | .030 | .075 | .205 | .522 | .308 | .811 | -.151 | .308 | .048 |
| 17. Average Clustering Coefficient _t_ | .201 | .223 | 0 | 1 | .011 | .043 | .148 | .040 | -.126 | -.007 | -.348 | -.062 | -.059 |
| 18. Standard deviation of Clustering Coefficient _t_ | .056 | .123 | 0 | .707 | .034 | .034 | -.038 | .347 | -.027 | .283 | -.019 | .481 | -.028 |
| 19. Average Genre Consistency _t_ | .422 | .177 | 0 | 1 | .005 | .006 | -.123 | -.036 | -.026 | -.016 | .071 | .008 | .011 |
| 2. Standard deviation of Genre Consistency _t_ | .053 | .097 | 0 | .707 | .045 | .071 | .056 | .447 | .011 | .329 | -.055 | .458 | -.014 |
| 21. Average BPM Consistency _t_ | .291 | .192 | 0 | 1 | -.001 | -.002 | -.009 | -.034 | -.055 | -.057 | .028 | -.023 | -.002 |
| 22. Standard deviation of BPM Consistency _t_ | .054 | .105 | 0 | .707 | .035 | .057 | .065 | .424 | .007 | .308 | -.058 | .422 | -.010 |
| 23. Average Key Consistency _t_ | .197 | .121 | 0 | 1 | .009 | .009 | -.063 | -.001 | -.047 | -.007 | .065 | .030 | -.024 |
| 24. Standard deviation of Key Consistency _t_ | .036 | .082 | 0 | .707 | .032 | .049 | .037 | .324 | -.004 | .240 | -.036 | .356 | -.019 |
| 25. Average Number of released tracks _t_ | 38.611 | 39.769 | 0 | 345 | -.023 | -.023 | -.029 | -.044 | .102 | .005 | -.052 | -.032 | .076 |
| 26. Standard deviation of Number of released tracks _t_ | 8.486 | 18.208 | 0 | 242.538 | .025 | .039 | .051 | .368 | .065 | .361 | -.066 | .389 | .013 |

| **Variable** | **10** | **11** | **12** | **13** | **14** | **15** | **16** | **17** | **18** | **19** | **20** | **21** | **22** | **23** | **24** | **25** |
| --- | --- | --- | --- | --- | --- | --- | --- | --- | --- | --- | --- | --- | --- | --- | --- | --- |
| 11. Average Indegree Reciprocity _t_ | .043 |  |  |  |  |  |  |  |  |  |  |  |  |  |  |  |
| 12. Standard deviation of Indegree Reciprocity _t_ | .180 | .441 |  |  |  |  |  |  |  |  |  |  |  |  |  |  |
| 13. Average Outdegree Reciprocity _t_ | .026 | .168 | .097 |  |  |  |  |  |  |  |  |  |  |  |  |  |
| 14. Standard deviation of Outdegree Reciprocity _t_ | .138 | .112 | .274 | .436 |  |  |  |  |  |  |  |  |  |  |  |  |
| 15. Average Betweenness Centrality _t_ | .053 | .472 | .214 | .171 | .107 |  |  |  |  |  |  |  |  |  |  |  |
| 16. Standard deviation of Betweenness Centrality _t_ | .258 | .186 | .501 | .074 | .273 | .376 |  |  |  |  |  |  |  |  |  |  |
| 17. Average Clustering Coefficient _t_ | -.007 | -.069 | -.011 | .035 | .029 | -.128 | -.017 |  |  |  |  |  |  |  |  |  |
| 18. Standard deviation of Clustering Coefficient _t_ | .169 | -.018 | .156 | -.004 | .159 | -.050 | .277 | .248 |  |  |  |  |  |  |  |  |
| 19. Average Genre Consistency _t_ | -.013 | -.018 | -.010 | -.027 | -.022 | -.043 | -.027 | .009 | .006 |  |  |  |  |  |  |  |
| 20. Standard deviation of Genre Consistency _t_ | .202 | .011 | .223 | .007 | .199 | .011 | .359 | .022 | .422 | .120 |  |  |  |  |  |  |
| 21. Average BPM Consistency _t_ | -.031 | -.055 | -.046 | -.039 | -.055 | .009 | -.039 | -.025 | -.044 | .200 | .058 |  |  |  |  |  |
| 22. Standard deviation of BPM Consistency _t_ | .197 | .002 | .201 | .005 | .181 | .020 | .354 | .014 | .379 | .020 | .647 | .178 |  |  |  |  |
| 23. Average Key Consistency _t_ | -.010 | -.035 | .002 | -.046 | -.012 | -.083 | -.015 | -.004 | .015 | .342 | .131 | .291 | .121 |  |  |  |
| 24. Standard deviation of Key Consistency _t_ | .142 | .003 | .172 | -.002 | .143 | -.008 | .261 | .010 | .309 | .107 | .644 | .143 | .618 | .347 |  |  |
| 25. Average Number of released tracks _t_ | .000 | .077 | .012 | .082 | .010 | .138 | .001 | -.037 | -.036 | .181 | -.071 | -.092 | -.085 | -.344 | -.106 |  |
| 26. Standard deviation of Number of released tracks _t_ | .215 | .051 | .257 | .047 | .240 | .068 | .387 | .006 | .361 | .050 | .471 | -.035 | .403 | -.033 | .365 | .205 |
